# A new species of *Notodiaptomus* from the Amazon basin (Crustacea, Copepoda, Calanoida, Diaptomidae)

**DOI:** 10.3897/zookeys.678.5978

**Published:** 2017-06-06

**Authors:** Daniel Previattelli, Gilmar Perbiche-Neves, Carlos E. F. da Rocha

**Affiliations:** 1 Universidade de São Paulo, Departamento de Zoologia, IB, Rua do Matão, travessa 14, número 101, Cidade Universitária, São Paulo, SP, Brazil; 2 Centro de Ciências da Natureza, Universidade Federal de São Carlos, Buri, SP, Brazil

**Keywords:** Amazonian Region, Brazil, Neotropical, pollution, reservoirs, Tocantins River, Xingu River

## Abstract

A new Diaptomidae species is presented from the Neotropical region. It was found in two Amazonian lakes, Ressaca and Arapujá, both in Pará State, Brazil. The lakes are 400 km apart and threatened by the building of reservoirs for hydropower generation and pollution by human settlements. The new species resembles *N. paraensis* Dussart & Robertson, 1984, but it can be distinguished from this species and other congeners in having a special process on the fifth leg basis of the male, by the place of insertion of lateral spine in the last segment of right P5 of male, the shape and relationship between length and width of segments of male and female P5 exopodite 2 with stout inner process bearing short setules and outer small spine, exopodite 3, with two terminal setae, outer smaller; endopodite 1-segmented with one subterminal seta and oblique comb of spinules, the presence of a line of dorsal spinules at the distal margin of thoracic somites in both sexes. A brief comparison with other *Notodiaptomus* species is presented in the discussion.

## Introduction

Among freshwater copepods, the family Diaptomidae is remarkable by its high endemism and high diversity. For example, no diaptomid species is shared between North and South America (Suárez-Morales et al. 2005; Perbiche-Neves et al. 2014). The endemicity of the South American species stands out for its high species richness, and is directly associated with the area of ecoregions (Perbiche-Neves et al. 2014).

Studies have been developed in Central and South America regarding biogeographic pattern and ecology as well as the description of new species (e.g. Paggi 2011, Perbiche-Neves et al. 2013), and the geographic distribution being constantly revised (Suárez-Morales et al. 2005; Santos-Silva 2008; Previattelli et al. 2013). There are also many problems concerning taxonomy of these organisms, such as the existence of synonyms and misidentifications. Despite of these advances, large portions of the continent are still poorly known, a situation that persists since first pointed out by Brandorff (1976), and reaffirmed by Santos-Silva (2013).

Moreover, the diversity of Diaptomidae is greatly biased by the concentrated investment of time of the researchers in certain areas, resulting in a distribution that has to do more with the distribution of the taxonomists than the true biogeographic patterns of the organisms (Previattelli et al. 2013). Even at present we still find new species in remote areas such as the Xingu and Tocantins river basins, nowadays the target of large reservoirs construction for hydropower generation. Other examples of gap areas in South America are reported in Perbiche-Neves et al. (2014).

The distribution of the species among Diaptomidae genera is uneven, with the genus *Notodiaptomus* Kiefer, 1936 being by far the most specious, with 39 of the 94 known species. Twenty-four of these species occur in Brazil (Santos-Silva 2008, 2013). During studies on two Amazonian lakes (Ressaca and Arapujá), located in Pará State, Brazil, a new species of *Notodiaptomus*, *Notodiaptomus nelsoni* sp. n., has been identified. The lakes are 400 km apart and threatened by building of reservoirs for hydropower generation and pollution by human settlements. A detailed description of a new species of *Notodiaptomus* is presented below.

## Materials and methods

Samples were collected using plankton nets of 60 µm mesh size and preserved in 70% alcohol. Vouchers containing ten females and ten males were deposited at the Museu de Zoologia da Universidade de São Paulo (MZUSP) and at the Instituto Nacional de Pesquisas da Amazônia (INPA).

Males and females were dissected using mounted entomological pins, and the most important structures drawn using a microscope equipped with drawing tube. Lacto-phenol added with glycerine was used as a mounting medium for these temporary preparations on slides. The morphologic terminology employed was according to Huys and Boxshall (1991), Santos-Silva et al. (1999), and Previattelli and Santos-Silva (2007).

### Abbreviations used

**Th1–Th6** thoracic somites 1 to 6

**Ur** Urosome somites

**GS** Genital double somite

**A1** Antennule

**A2** Antenna

**Md** Mandible

**Mxl** Maxillule

**Mx** Maxilla

**Mxp** Maxilliped

**P1–P4** First to fourth swimming legs

**P5** Fifth leg

**Enp** Endopod

**Exp** Exopod

Exp-1 (-2, -3) refer to the first, second and third segments of leg exopods. The abbreviation Enp-1 (-2, -3) refers to segments 1-3 of the leg endopods.

The previously called “vestigial seta” found in segments of the A1 is present and follows the same pattern as the other members of the genus. The term is not used since it is not clear whether if it is a proper armature element (a reduced seta), as proposed by Santos-Silva et al. (1999).

## Taxonomy

### Family Diaptomidae Baird, 1850

#### Subfamily Diaptominae Kiefer, 1932

##### Genus *Notodiaptomus* Kiefer, 1936

###### Notodiaptomus nelsoni

Taxon classificationAnimaliaCalanoidaDiaptomidae

Previattelli, Perbiche-Neves & Rocha sp. n.

http://zoobank.org/DE18F426-3DCE-4789-B8E7-3515F33AD6D2

Figures 1 , 2 , 3 , 4 , 5 , 6 , 7 , 8 , 9

####### Material examined.

*Holotype*. One male, entire, alcohol + glycerine (MZUSP30604), Arapujá Lake, 3°12'54"S, 52°11'28"W, Xingu River Basin, in front of Altamira, Pará State, 21 October 1997, Jansen Zuanon col. *Paratypes*. Ten males and ten females, entire, alcohol + glycerine (MZUSP30605), one male and one female dissected and mounted on slides in glycerine (MZUSP30606), Arapujá Lake, Xingu River, Altamira city, Pará State, 21 October 1997, Jansen Zuanon col. *Additional material*. Males, females and copepodids from Ressaca Lake, Tocantins River Basin, 5°11'36"S, 49°15'45"W, June 1983, Pedro Mera col.

**Figure 1.**
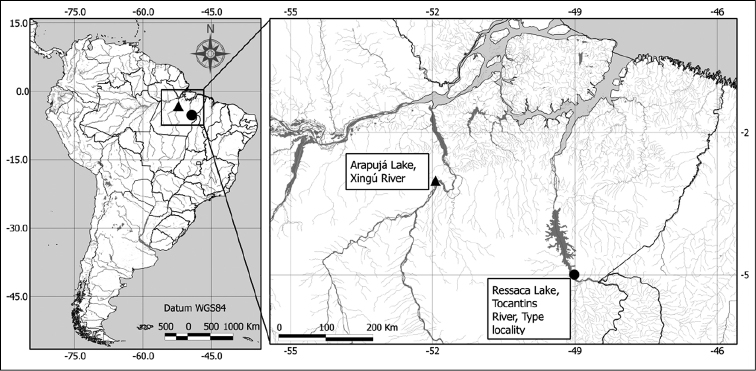
Study area with records of *Notodiaptomus nelsoni* sp. n., at Arapujá Lake and Ressaca Lake.

####### Etymology.

Named in honour of Dr. Edinaldo Nelson dos Santos Silva (Instituto Nacional de Pesquisas da Amazônia, INPA, Manaus, Brazil), for his invaluable contributions to taxonomy and ecology of the Diaptomidae.

####### Diagnosis.

**Figure 2.**
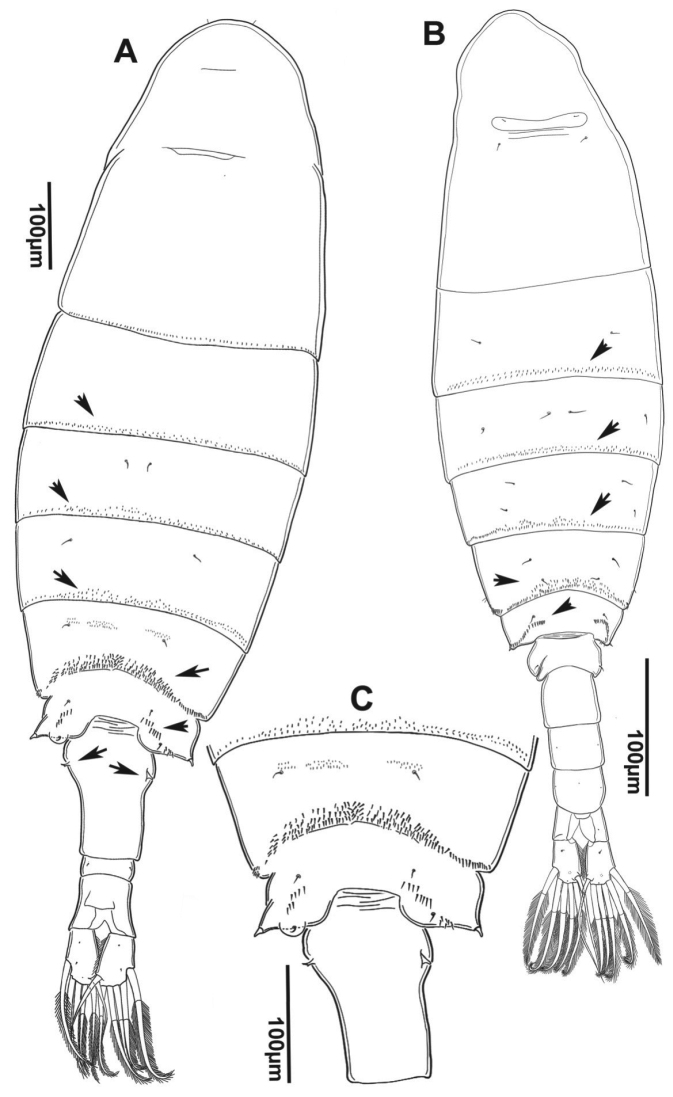
*Notodiaptomus nelsoni* sp. n. **A** Habitus of female, dorsal **B** Habitus of male, dorsal **C** Detail of last pedigerous somites and genital double somite in female, dorsal. Arrows points lines of spinules on dorsal surface and sensilla at GS.

*Male* (Figures 2B, 10A). Length excluding caudal setae (mean ± SD of ten specimens: 907±60 µm from Ressaca Lake; 859±18 µm from Arapujá Lake). Transverse, narrow nuchal organ ahead cephalic suture, with pair of small sensilla internally and another larger pair of sensilla adjacent to suture (Figure 2B). Rows of fine dorsal spinules along posterior margins of Th2 to Th6 (Figure 2B). Pair of lateral wings symmetrical, with curved row of spinules on each wing. First segment of left A1 with patch of spinules. Segment 13 of right geniculate antennule produced into well-developed, spinous process. Segments 15 and 16 with small process each. Pair of P5 asymmetrical, both with rudimentary, unarmed praecoxae and coxae bearing posterior conical process projecting over basis. Right P5 bearing outgrowth on posterior basal surface with deep oblique groove with minute tubercles along edge; semicircular lamella on inner margin of basis covered with fine setae; exopod 2-segmented, exp-1 with posterior distal margin produced into pair of conical outgrowths ending as blunt tip; Exp-2 with lateral outer spine basally placed at the distal third and terminal claw strong and curved in two planes. Right P5 endopod one-segmented partially fused to the basis, anteriorly.

*Female* (Figure 2A). Length excluding caudal setae (mean of ten specimens: 1,063±35 µm from Ressaca Lake; 933±31 µm from Arapujá Lake). Rows of dorsal spinules along posterior margins of Th2 to Th6 (Figure 2A, C). Posterolateral wings of Th5 asymmetrical; left wing produced into a semicircular expansion with sensilla at tip. GS slightly asymmetrical. Left spiniform sensilla with bifid apex (Figure 2A, C). P5 with fused intercoxal plate, making legs be closely placed. Dorsal expansions of coxa symmetrical and less than ¼ of segment; strong spiniform sensilla at apex of expansions. Lateral seta of basis reaching from half and 2/3 of length of Exp1; Exp three-segmented, Exp 1 unarmed, Exp2 with stout inner process bearing short setules and outer small spine, not fused to segment. Exp3 reduced, with two unequal spines, the outer smallest and reaching more than 1/2 length of inner spine. Enp one-segmented, with no sutures; length reaching 2/3 of Exp1 at least (Figure 9D, E).

####### Description - male

(Figure 2B). Length of holotype, excluding caudal setae, 1,075 µm.

*Prosome*. Male shorter than female. Maximum width of prosome (275 µm) at Th1. Rostrum asymmetrical (Figure 8E). Cephalosome with nuchal organ just anterior to suture level; two pairs of sensilla next to lateral borders. Prosome 5-segmented. Th5 and Th6 fused, with suture line represented by rows of spinules (Fig. 2B). Lateral wings little developed, each with small spiniform sensilla at apex, similar in size and shape to spinules composing a dorsal row (Figure 2B).

*Urosome* (Figures 2B, 10A). Consisting of four somites plus anal segment, genital somite asymmetrical, with left genital aperture at the middle portion; pair of sensilla at similar regions on both sides of the segment. Ur1 to 3 longer than wide, with small pores laterally; small anal operculum at Ur4.

*Caudal rami* (Figure 2B). Symmetrical, longer than wide, with six plumose setae at posterior portion; innermost setae slender and smooth; two dorsal sensilla and setules along inner margin.

**Figure 3.**
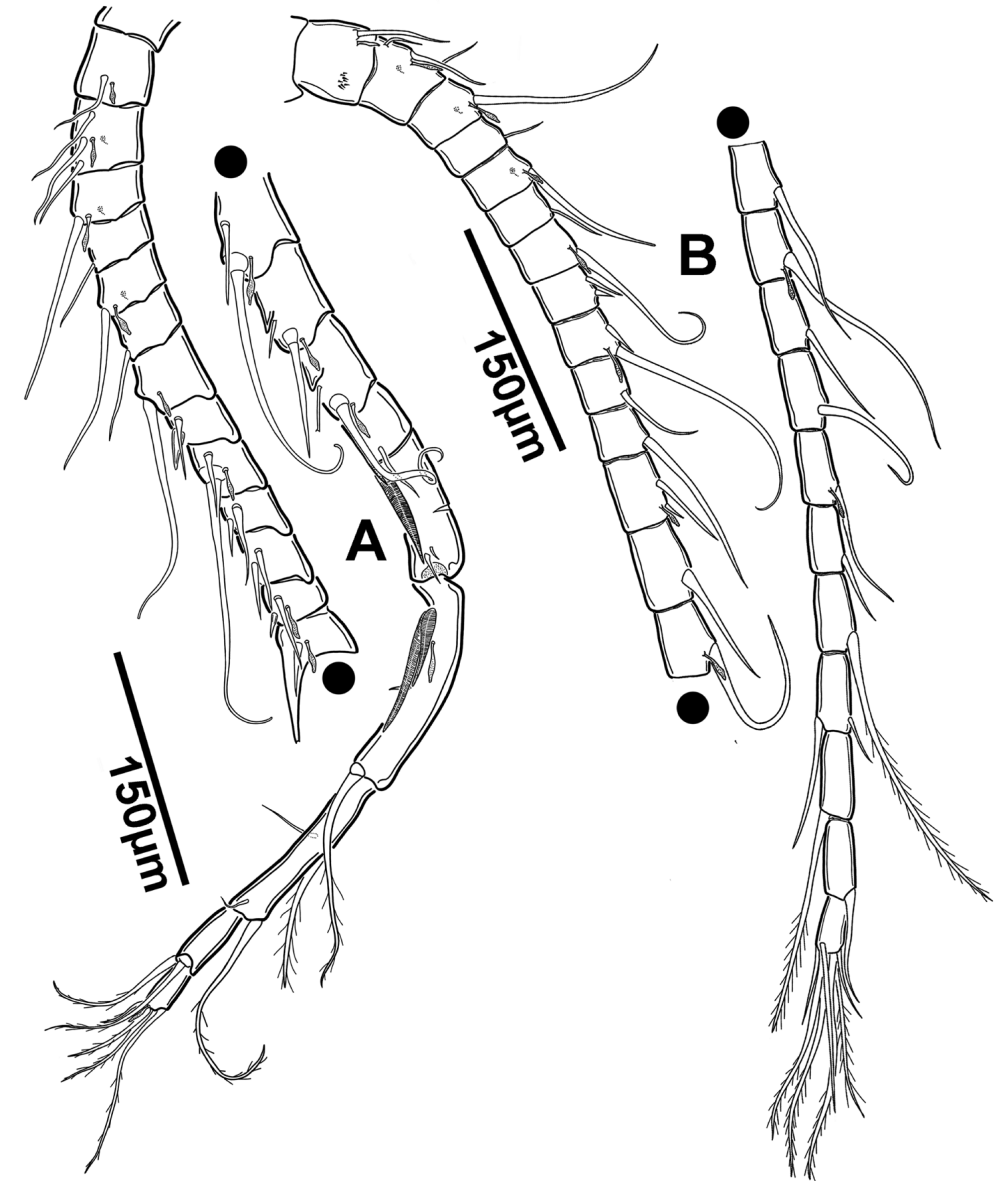
*Notodiaptomus nelsoni* sp. n. **A** Right geniculate antennule of male **B** Left antennule of male.

**Figure 4.**
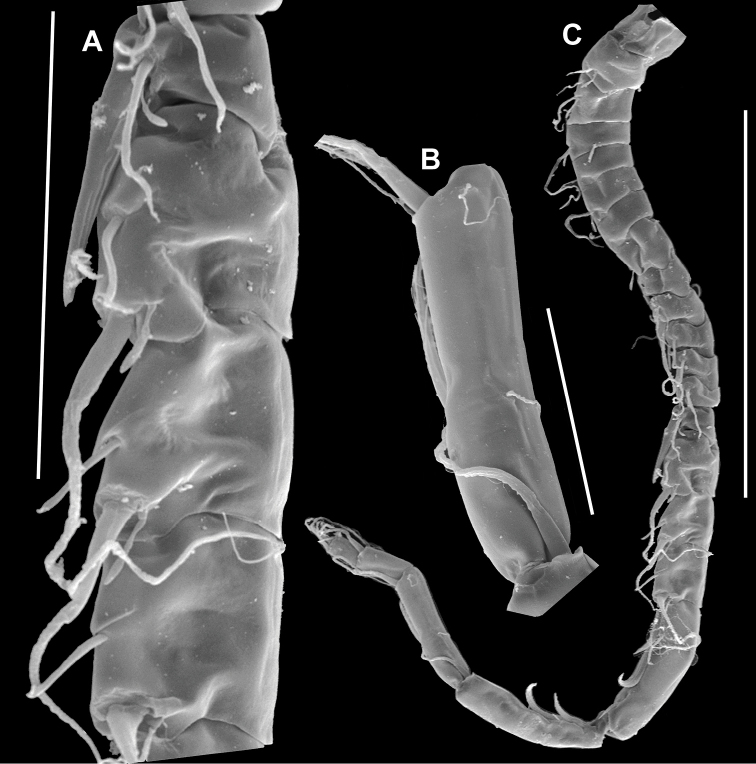
*Notodiaptomus nelsoni* sp. n. male. **A** Right geniculate A1, segments 13–16 (100 µm), arrow point the spinous process at segment 13 of antennule **B** Right geniculate A1, segment 20 (50 µm) **C** Complete right geniculate A1 (300 µm).

*Antennules* (Figures 3A, B, 4A–C). Asymmetrical, extending beyond prosome but not extending past the distal portion of the Ur3. Ancestral segments II–IV, XXI–XXIII, XXIV–XXV and XXVII–XXVIII completely fused. Tip of setae on segments 3 (V), 7 (IX), 9 (XI) and 14 (XVI) blunt.

*Right antennule* (Figures 3A, 4A–C). Composed by 22 segments; fusion pattern and armature presented in Table 1. Sensilla present at segments 2 (III), 3 (V), and 5 (VII). Conical seta of segment 8 (X) longer and more robust than the one at segment 12 (XIV). Modified seta on segment 10 1/3 smaller than modified seta on segment 11. Seta-like aesthetasc present at segments 17 and 18 (XIX, XX). Tips of large setae on segments 3, 7, 9 and 14 blunt. Segment 19 with one very long and one short setae. Modified seta forming strong process on segment 13. Segments 15 and 16 with small spinous process.

**Table 1.** Segmentation and armament of the antennules in both adult male and female of *Notodiaptomus nelsoni* sp. n. A, ancestral segments according to Huys and Boxshall (1991); ae, aesthetasc; ms, modified seta; N, segments in adults; p, process; s, seta; vs, vestigial seta.

A	N	Male right antennule	N	Male left antennule	Female antennules
I	1	1s + 1ae	1	1s + 1ae	1s + 1ae
II	2	3s + 1ae	2	3s + 1ae	3s + 1ae + 1vs
III					
IV					
V	3	1s + 1ae	3	1s + 1ae	1s + 1ae + 1vs
VI	4	1s	4	1s	1s
VII	5	1s + 1ae	5	1s + 1ae	1s + 1ae + 1vs
VIII	6	1s	6	1s	1s
IX	7	1s + 1ae	7	1s + 1ae	1s + 1ae
X	8	1s + 1cs	8	1s + 1cs	1s + 1cs
XI	9	2s + 1ae	9	2s + 1ae	2s + 1ae
XII	10	1s + 1ms	10	1s	1s
XIII	11	1s + 1ms	11	1s	1s
XIV	12	1s + 1ae + 1cs	12	1s + 1ae + 1cs	1s + 1ae + 1cs
XV	13	1s + 1ae + 1ms	13	1s	1s
XVI	14	2s + 1ae	14	1s + 1ae	1s + 1ae
XVII	15	2s + 1ae + 1p	15	1s	1s
XVIII	16	2s + 1ae + 1p	16	1s + 1ae	1s + 1ae
XIX	17	2s + 1ms	17	1s	1s
XX	18	1s + 1 ms	18	1s	1s
XXI	19	2s + 1ae + 2ms	19	1s + 1ae	1s + 1ae
XXII			20	1s	1s
XXIII			21	1s	1s
XXIV	20	4s	22	2s	2s
XXV			23	2s	2s
XXVI	21	2s	24	2s	2s
XXVII	22	4s + 1ae	25	4s + 1ae	4s + 1ae
XXVIII					

*Left antennule* (Figure 3B). 25-segmented; armature of segments presented in Table 1. Tips of large setae on segments 3, 7, 9 and 14 blunt, as in right antennule. Seta inserted ventrally on segment 24 (XXVI).

**Figure 5.**
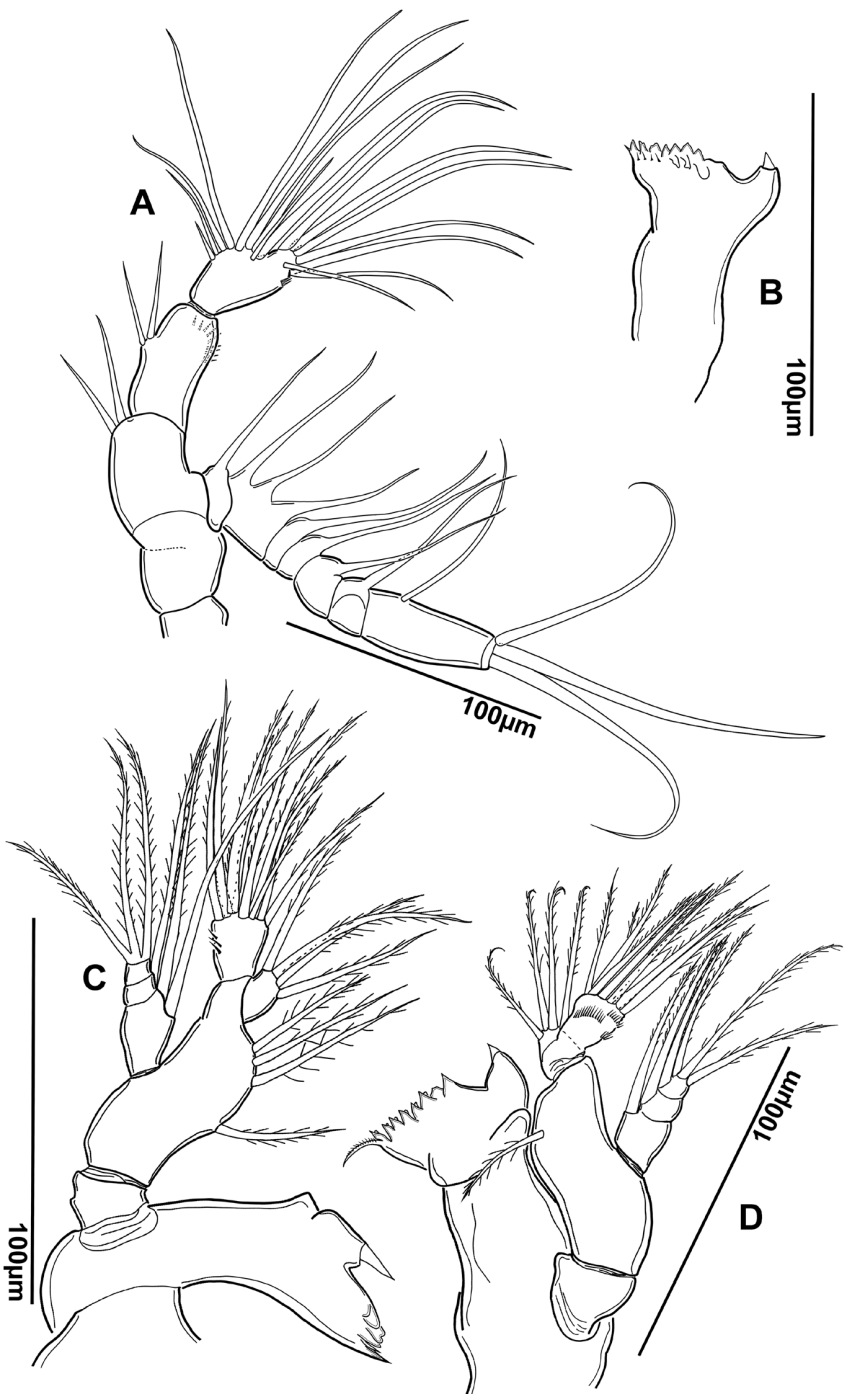
*Notodiaptomus nelsoni* sp. n. male. **A** Right antenna **B** Coxal gnathobase of right mandible **C** Mandible, ventral view **D** Mandible, dorsal view.

*Antenna* (Figure 5A). Biramous; coxa presenting one inner seta. Basis with two setae inserted posteriorly. Exopod 8-segmented; second (II–IV) and penultimate (IX–X) segments compounded with regions of discontinuous cuticle surface; penultimate segment elongated; distal segment small, with three long, apical setae. Endopod 2-segmented; outer margin of first segment ornamented with one patch of spinules (approx. 15); inner margin with two setae and pore between patch of spinules and setae; second segment bilobed, with groove between lobes; outer lobe with seven (eight visible on Fig. 5A) marginal setae and one group of spinules on dorsal/outer margin; inner lobe with eight distal setae.

*Mandible* (Figure 5B, C, D). Gnathobase strongly sclerotized; gnathal blade with six multicuspid teeth and distal sub-triangular tooth. Basis with inner seta. Mandibular palp biramous. Basis of the palp with four inner setae (three distal). Exp with four segments, setal formula 1, 1, 1, 3. Enp 2-segmented; first segment with distal lobe bearing four setae; second segment with nine distal setae and three rows of spinules on posterior margin.

**Figure 6.**
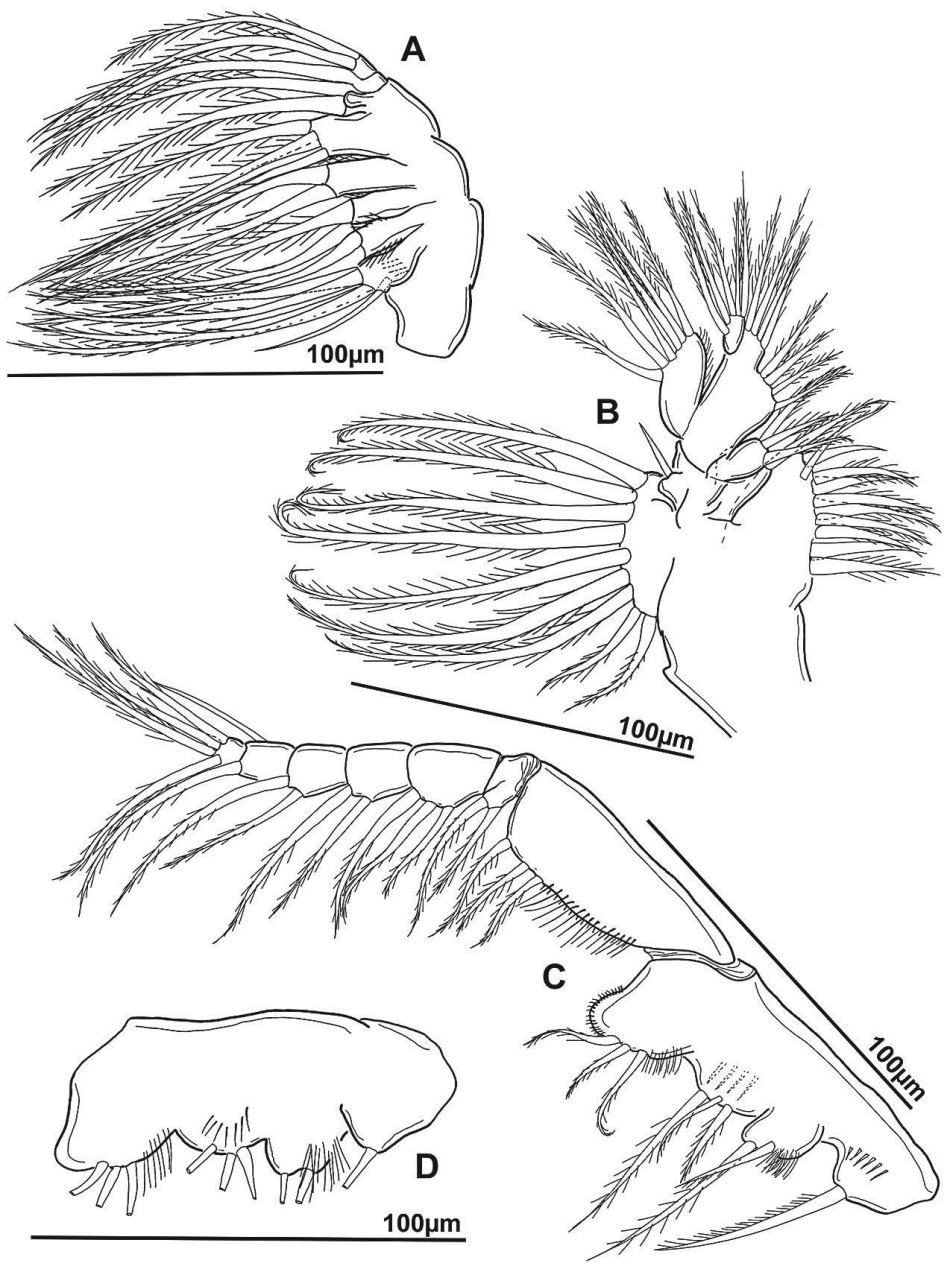
*Notodiaptomus nelsoni* sp. n. male, mouth parts. **A** Left maxilla **B** Maxillule **C** Frontal view of maxilliped **D** Caudal view of maxilliped.

**Figure 7.**
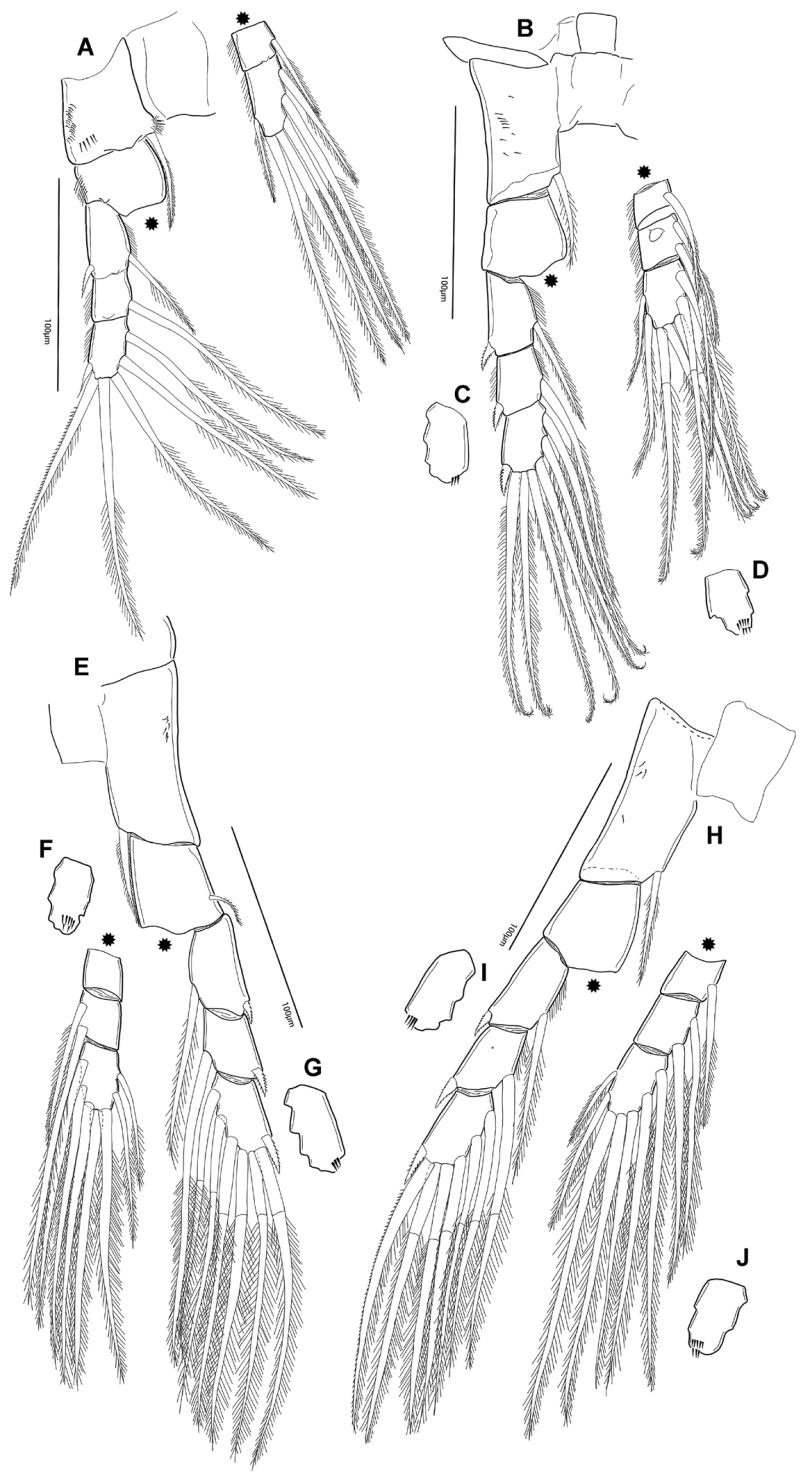
*Notodiaptomus nelsoni* sp. n. Male **A** P1, anterior view **B** P2 **C, D**. Detail of caudal view of last segment of exopodite (**C**) and endopodite (**D**) of P2 **E** P3 **F, G** Detail of caudal view of last segment of endopodite (**F**) and exopodite (**G**) of P3 **H** P4 **I, J** Detail in caudal view of last segment of exopodite (**I**) and endopodite (**J**).

*Maxillule* (Figures 6B, 7A). Coxal epipodite with nine setae and row of spinules on distal surface. Coxal endite with four distal setae. Outer seta representing basal exite present; four setae on proximal basal endite, distal basal endite with four setae. Endopod two-segmented; with three setae on margin of proximal segment, and five distal setae on second segment. Exopod with six distal setae.

*Maxilla* (Figure 6A). Proximal praecoxal endite with five setae and one spine (setules present on these setae but not figured here); distal praecoxal endite with three setae: proximal and distal coxal endites each with three setae; allobasis with four setae; free endopod with five setae in total.

*Maxilliped* (Figures 6C, D). First syncoxal endite represented by one seta with row of spinules at base; second to fourth syncoxal endites from proximal to distal with 2, 3, 3 setae, distal angle of syncoxa extended into lobe with row of small spinules; basis with three setae, double row of setules proximally; endopod six-segmented, with 2, 3, 2, 2, 1+1, 4 setae.

*P1* (Figure 7A). Coxa with inner seta inserted distally, adjacent to a small round expansion ornamented with setules; outer margin bearing one patch of setules and one line of spinules. Basis with setules line at outer margin. Exp 3-segmented; setules along inner margin of the first segment, and outer margin of the second and third segments. Exp-3 spine with serrate margin at external side, and internal surface of Exp-3 without line of spinules. Enp 2-segmented; setules present along all outer margins.

**Figure 8.**
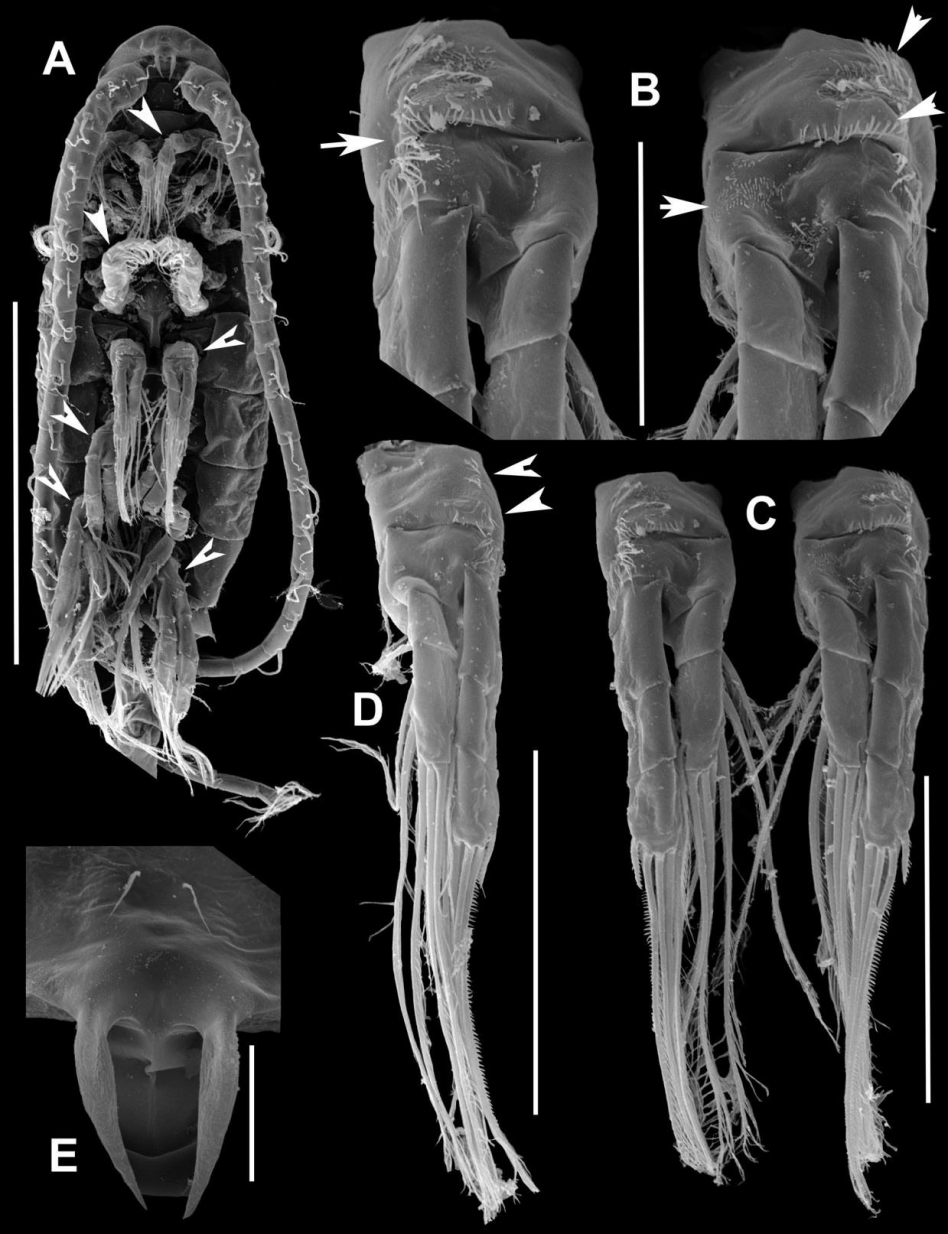
*Notodiaptomus nelsoni* sp. n. male. **A** Habitus, ventral view (400 µm), arrows point to antenna, maxilliped, P1, P2, P3, P4 **B** Frontal view with detail of coxa and basis of P2 (50 µm), arrows point to small patches of spinules **C** P2 (100 µm), arrows point to small patches of spinules **D** Frontal view of left P4 (100 µm), arrows point to small patches of spinules **E** Detail of rostrum filaments (20 µm).

*P2* (Figures 7B–D, 8B, C). Coxa with inner seta inserted distally. Posterior surface ornamented with a patch of spinules. Basis with no setal element or ornaments. Exp 3-segmented, with one outer spine each; setules along inner margin of the first, inner and outer of the second segment. Anterior surface of Exp-3 with distal row of spinules. Enp 3-segmented. Schmeil’s organ present on posterior surface of segment 2. Setules along outer margin of all segments. Enp-3 anterior surface with two distal rows of spinules.

*P3* (Figure 7E, F, G). Coxa with inner seta inserted distally. Posterior surface ornamented with small spinules patch. Basis with no setal element or ornaments. Exp with setules along inner margin of the first and second segments. Anterior surface of Enp-3 with one line of spinules at distal part. Remaining characters like P2.

*P4* (Figures 7H–J, 8D). Coxa with inner seta inserted distally. Posterior surface ornamented with small patch of spinules. Basis with one seta inserted at the posterior/outer margin. Exp without setules along inner or outer margins. Anterior surface of Exp-3 and Enp-3 with one and two lines of spinules at distal part, respectively. Armature formula of all legs represented in Table 2.

**Table 2.** Setae and spine formula for swimming legs of *Notodiaptomus nelsoni* sp. n.

	Coxa	Basis	Exopod	Endopod
P1	0-1	0-0	I-1; 0-1; I,I,4	0-1; 1,2,3
P2	0-1	0-0	I-1; I-1; I,I,5	0-1; 0-2; 2,2,3
P3	0-1	0-0	I-1; I-1; I,I,5	0-1; 0-2; 2,2,3
P4	0-1	1-0	I-1; I-1; I,I,5	0-1; 0-2; 2,2,3

**Figure 9.**
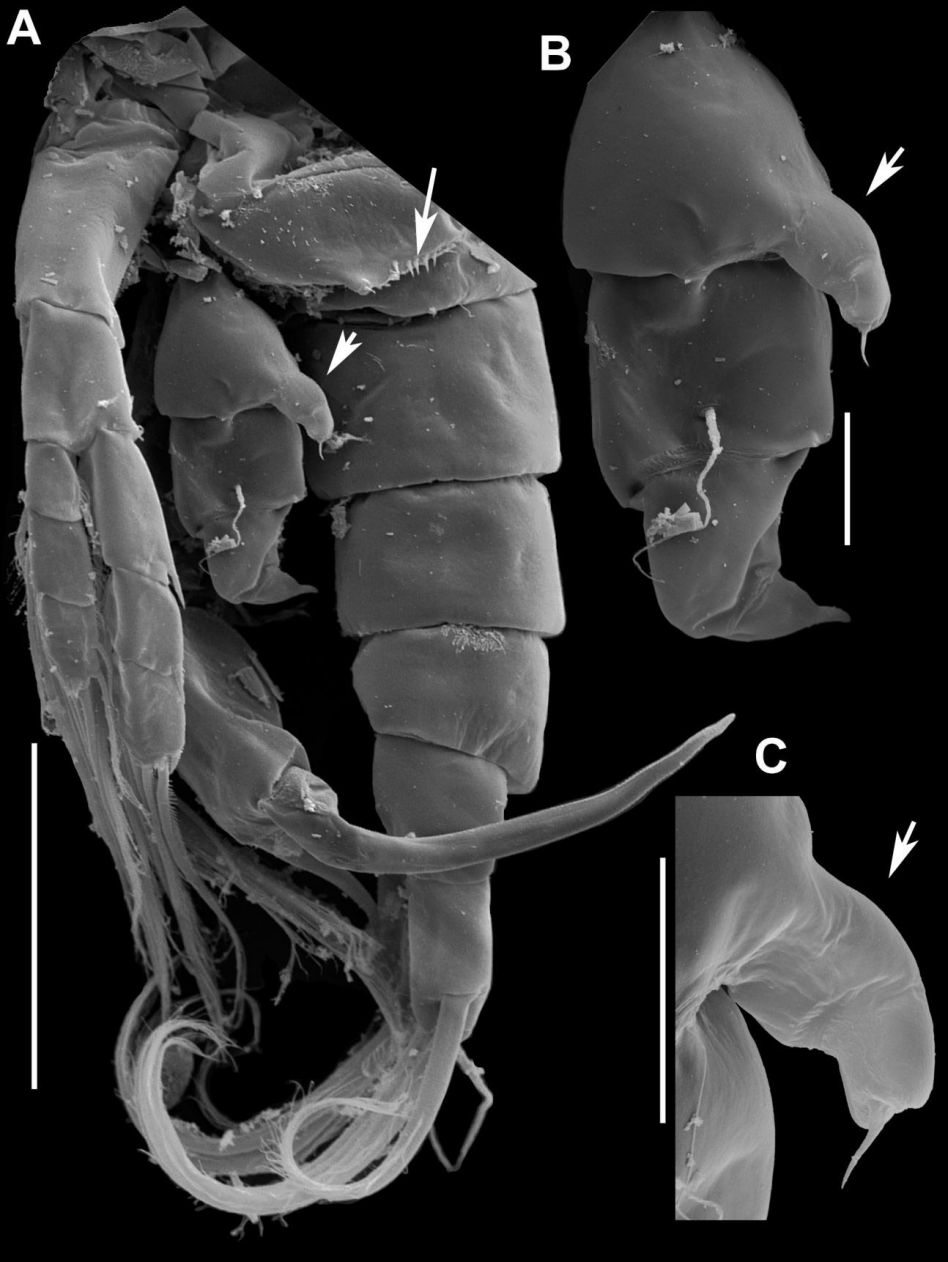
*Notodiaptomus nelsoni* sp. n. male. **A** P4, P5, and Ur (100 µm), arrow points to the line of spinules on the distal margin of pediger 5, to the process at the basis of left P5, and to the exopodite 2 of right P5 **B** Left P5 (20 µm), arrow points to the expansion in the basis of left P5 **C** Detail of the conical process in the basis of left P5 (20 µm), arrow points to the expansion in the basis of left P5.

**Figure 10.**
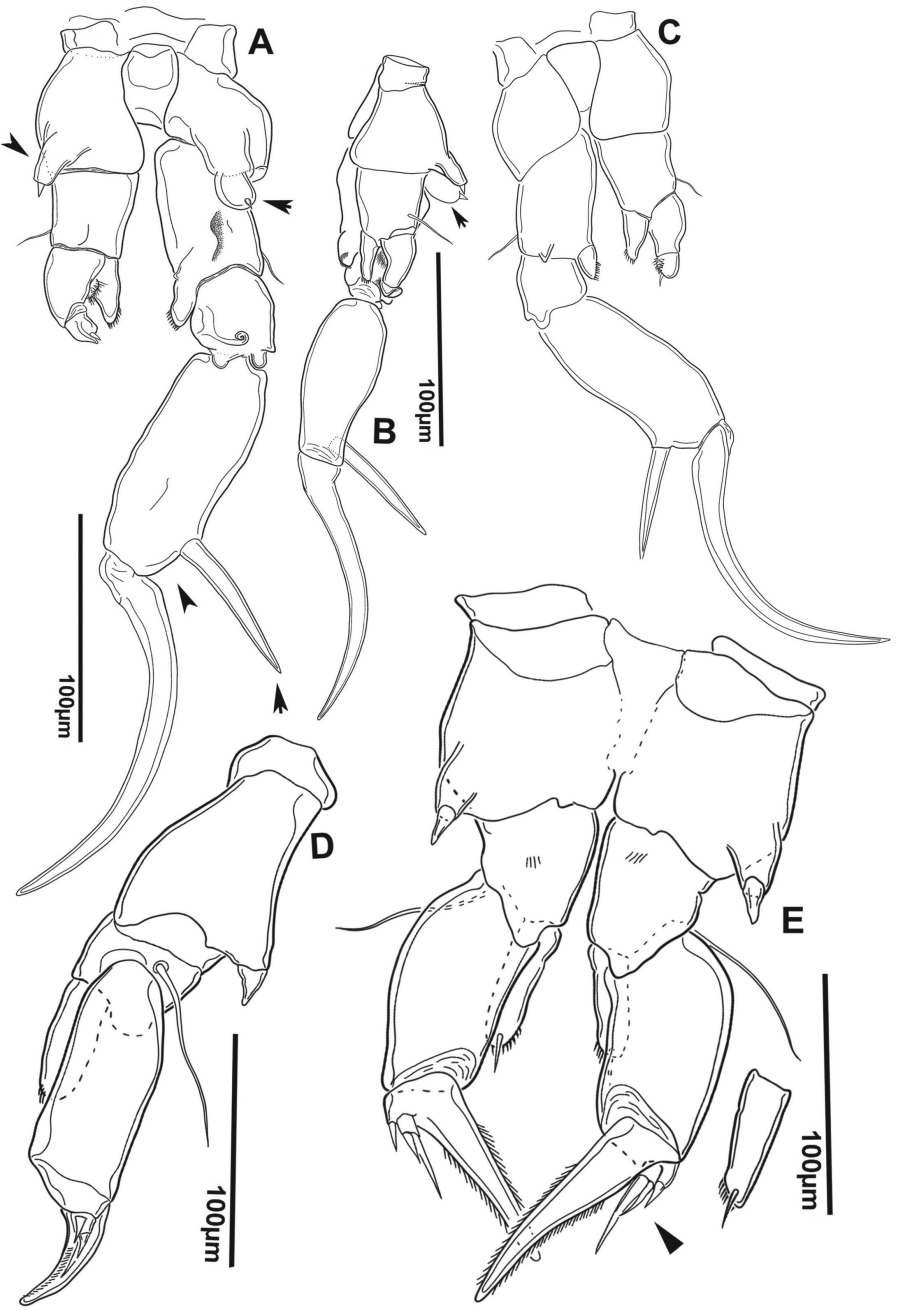
*Notodiaptomus nelsoni* sp. n. **A, B** Fifth leg (P5) of male, caudal (**A**) and in a lateral view (**B**), arrows point to process expansion at basis in left and right P5, and the short distance between lateral spine and terminal claw **C** P5 of male in anterior view **D** P5 of female in latero-caudal view **E** P5 of female in anterior view, arrow points to the external seta of exopodite 2.

*P5* (Figures 9A–C, 10). P5 asymmetrical and biramous; rudimentary praecoxa present; intercoxal plate (coupler) longer than wide.

*Right P5* (Figures 9A–C, 10A). Coxa with conical process projecting over basis with spiniform sensilla acute at tip (Figure 7C). Basis with lateral seta inserted at distal part. Outgrowth on posterior basal surface with deep oblique groove ornamented with minute tubercles along edge, Enp one-segmented, as endopodal lobe with comb of spinules on inner anterior surface. Exopod2-segmented; first segment with acute sclerotized outgrowth on distal margin, posteriorly; second segment with curved ridge on posterior surface, lateral spine on distal third of segment; terminal claw strong and curved proximally, with row of spinules along inner margin. Endopodal lobe with comb of spinules on inner anterior surface.

*Left P5* (Figures 9A–C, 10). Well developed, reaching distal margin of right Exp-1. Coxa with conical process projecting over basis with spiniform sensilla at tip, very similar to the one at right. Basis with seta on outer margin; inner margin slightly curved; Exopod bisegmented. Exp-1with convex outer margin; inner margin with rounded process bearing setules. Exp-2 with semicircular process bearing setules proximally on inner margin, inner small spine and apical spiny process.

####### Description – female

(Figure 2A). Length, excluding caudal setae 1,670 µm. Maximum width at distal margin of the Th1, with 510 µm. Body larger than male. Prosome. Rostrum symmetrical, with paired filaments, right broader in mid part than left. Prosome with incomplete dorsal suture separating Th5 and Th6, suture marked by spinules dorsally and laterally. Posterolateral wings of Th6 small and slightly asymmetrical; left wing slightly larger than right and with semicircular shaped protrusion on distal margin; right wing elongate; both lateral wings with spiniform sensilla (Figure 2A, C).

*Urosome* (Figure 2A, C). Ur3-segmented; GS inflated anteriorly and nearly symmetrical, longer than all the other somites combined; anal segment with weakly developed operculum; caudal rami symmetrical with setules along inner margin; genital area, located centrally with prominent lateral processes on opercular pad.

*A1*. Symmetrical, 25-segmented; similar to left A1 of male (Figure 3B); extending beyond half of the GS.

*P5* (Figure 9D, E). Symmetrical, coxa with conical process with sensilla at tip, basis subtriangular with long seta reaching beyond half of Exp-1 and bearing a small row of setules on anterior surface (Figure 9E). Exp-1 larger than second, Exp2 with stout terminal claw with denticles along lateral margins bearing short setules and outer small spine, not fused to segment. Exp-3 with two terminal setae, lateral smaller; enp 1-segmented with two setae and oblique comb of spines sub terminally on anterior surface. Number of armature elements as in Table 2.

## Discussion

The new species shares homologies with the genus *Notodiaptomus* and fits well the generic characters as presented by Santos-Silva et al. (1999). It is close to *Notodiaptomus paraensis* Dussart & Robertson, 1984, from which it differs by the presence of dorsal rows of spinules at the distal border of all prosomites in male (Fig. 2A, B), by the size of the conical process at the distal margin of the coxa in the male right and left P5 (Fig. 9A, B,C) , in female, the position of the lateral setules on GS (Figure 2A), as well as the shape and element configuration of the P5 exopodite 2 (Figure 9D, E).A more detailed comparison between these two species composes Table 3 that compiles morphological characters found, in which the new species is compared to the original description of *N. paraensis* (Dussart & Robertson, 1984) and the re-description from Santos-Silva et al. (1989).

**Table 3.** Comparison between differential characters between *N. nelsoni* sp. n. and *N. paraensis*, based on Dussart and Robertson (1984) and Santos-Silva et al. (1989).

Structures	*N. nelsoni* sp. n.	*N. paraensis*
**Male**		
Rostrum filaments	Filaments length at least three times longer than the width of the basis of these filaments	Filaments length no more than three times the width of the basis
Dorsal spinules at distal margin of thoracic somites	Multiple fine lines at distal margin	Without dorsal spinules
Last segment of right antennule	Without any process	With falciform process
Right P5	Well-developed (1.8 times longer than broad) projection at distal margin of coxa	Small projection at distal margin of coxa
Right P5	Lateral spine inserted at the distal third of Exp2	Lateral spine inserted at the middle of Exp2
Right P5	Exp2 up to two times longer than wide	Exp2 less to two times longer than wide
Left P5	Well-developed (1.7 times longer than broad) projection at distal margin of coxa	Small projection at distal margin of coxa
Left P5	Short sensilla at the top of projection at the distal margin of coxa, until three times longer than the width of the basis of this sensilla.	Longer sensilla at the top of projection at the distal margin of coxa, up to four times longer than the width of the basis of this sensilla.
**Female**		
Double genital segment	Lateral sensilla in the same position in a dorsal view.	Lateral sensilla in different positions on a dorsal view, right one located more anteriorly than left one.
Dorsal spinules at distal margin of thoracic somites	Multiple fine lines at distal margin	Without dorsal spinules
P5 Exp2	Two times longer than broad, short lateral seta, less than ¼ of the length of the segment of Exp3	1,5 times longer than broad, long lateral setae, reaching 2/3 of the length of Exp3

The new species also differs of other species from the *Notodiaptomus* genus sensu Santos-Silva (1999. Particularly, compared to the type, *Notodiaptomus deitersi* (Poppe, 1891), the new species differs by the segments 2 and 3 of male geniculate antennule, by the presence of dorsal spinules on thoracic somites, in the length proportions between lateral spine and terminal claw of male right P5, the length of the seta of the coxa of the female P5, etc. Compared to *Notodiaptomus henseni* (Dahl, 1894), this last seems longer than the new species, and about the structures they are different in the shape of lateral projections of genital segment of female, the length of lateral spine of right P5 of male. Compared to *Notodiaptomus amazonicus* (Wright, 1935), this species doesn’t have lines of spinules at dorsal surfaces of thoracic somites, both male and female are longer, the shape of genital segment of female differs from the new species, P5 right male of *N. amazonicus* have a notch at basis inner margin. Finally, our new species is also different from *Notodiaptomus nordestinus* (Wright, 1935) regarding the male and female P5 basis with small spinules in the inner margin and in coxa, as well as the dorsal surface of the thoracic somites.

The description of this new species increases the number of valid species of the genus accordingto Santos-Silva (2008) to 40. Phylogenetic studies are necessary to clarify relationships and rearrangements in the genus *Notodiaptomus*. Currently it is considered as an artificial and problematic one (Santos-Silva 2013), and a new phylogeny combining morphological and molecular characters should probably reduce the number of valid species in this genus.

The Amazon basin is the richest area in the Neotropical region concerning the presence of diaptomid species, due in large part to its area extension (Perbiche-Neves et al. 2014). It is expected that some new species will be described from this region, as well as in other large river basins in this zoogeographical area. Large tributaries of Amazonas River have high potential to contain new species, as often mentioned in the literature.

During the period in which the samples were obtained (1983 and 1997) until today, many changes have already occurred in some of the large tributaries of Amazonas River. Especially, we refer to the intense activities of deforestation (agriculture and livestock), and the construction of large reservoirs for hydropower generation. The new species was found in old samples collected before the intense human activity, and their presence today in the same places is not known, but its probable absence might indicate how strong such interference has been.

The results also emphasize the need of intense research in other rivers threatened by the already mentioned activities, such as the rivers Teles Pires, Tapajós, and Madeira, in Brazil, as well as in other countries of the Amazon basin, such as Peru, Ecuador, Colombia, Venezuela, etc.

## Supplementary Material

XML Treatment for Notodiaptomus nelsoni
